# Autoimmune antibodies and systemic inflammatory markers are prevalent and associated with cognition in individuals aged 90+

**DOI:** 10.1177/13872877251365560

**Published:** 2025-08-08

**Authors:** Ghasem Farahmand, Anne-Marie C Leiby, Jiaxin Yu, Aanan Ramanathan, Rojan Javaheri, Claudia H Kawas, Davis C Woodworth, Maria M Corrada, Tianchen Qian, S Ahmad Sajjadi

**Affiliations:** 1 Department of Neurology, University of California, Irvine, CA, USA; 2 Department of Statistics, University of California, Irvine, CA, USA; 3 Department of Neurobiology & Behavior, University of California, Irvine, CA, USA; 4 Institute for Memory Impairments and Neurological Disorders, University of California, Irvine, CA, USA; 5 Department of Epidemiology & Biostatistics, University of California, Irvine, CA, USA; 6 Department of Pathology, University of California, Irvine, CA, USA

**Keywords:** Alzheimer's disease, amyloid, autoimmunity, cognition, inflammation

## Abstract

**Background:**

While recent studies have found associations between markers of autoimmunity/inflammation and cognitive performance in individuals aged 60–90, these findings remain unexplored in individuals aged 90 and above.

**Objective:**

To examine the prevalence of autoimmune antibodies and raised inflammatory markers and their associations with cognition in participants aged 90 + .

**Methods:**

We included participants with serological testing from The 90+ Study, a community-based longitudinal study in southern California. For measures of autoimmunity, we evaluated antinuclear, antineutrophil cytoplasmic (ANCA), rheumatoid factor, double stranded DNA, antithyroglobulin, and thyroid peroxidase antibodies. For inflammatory markers, we examined interleukin-6 (IL-6) and erythrocyte sedimentation rate (ESR). To examine the relationship between autoimmune antibodies and inflammatory markers with cognitive performance, we ran linear mixed effects models.

**Results:**

Among 201 participants (mean age 94.8 years, 56.7% female, 93.5% white, and 4.5% with rheumatologic illness), autoimmune antibodies were positive in 70.2%. Also, among 142 participants with test results, elevated inflammatory markers were detected in 76.8%. Linear mixed effects model analyses revealed an association between higher levels of ANCA (*p* = 0.04), IL-6 (*p* = 0.01), and ESR (*p* = 0.01) and lower global cognitive scores. In a subset of participants with amyloid PET (*n* = 173), results remained significant even after accounting for amyloid burden.

**Conclusions:**

Autoimmune antibodies and raised inflammatory markers were highly prevalent in a community cohort of individuals aged 90 + . Our results suggest that increased prevalence of autoimmunity and inflammation might be associated with worse cognitive performance in this age group, independent of amyloid.

## Introduction

Incidence of dementia increases with advancing age and is at its highest in the tenth and eleventh decades of life.^1,2^ Alzheimer's disease pathology remains prevalent in individuals aged 90 + but other degenerative and vascular pathologies also become prevalent^3–7^ Aging is widely considered the most important risk factor for developing dementia and its related neuropathologies, but mechanisms through which the aging brain becomes susceptible to degeneration and cognitive impairment remain elusive.^1–3,8^ Studies of individuals across the life span suggest that serological markers of inflammation and autoimmunity also become more prevalent with advancing age.^9,10^ In many instances, the individuals harboring these inflammation markers and antibodies do not have the classical features of rheumatological disease or inflammation typically ascribed to these markers.^9–12^ There are suggestions in individuals younger than 90 that these markers of inflammation and autoimmunity might be related to cognitive impairment and certain neuropathologies, but such associations have not been studied in individuals aged 90+.^1,12^ At a more basic level, even the frequency of these markers in individuals aged 90 + is unknown since previous studies do not include individuals older than 90.

There is, therefore, an unmet need for establishing the frequency of these autoimmunity and inflammation markers in the 90 + age group and to study putative associations between these markers and cognitive impairment in this age group, that is the fastest growing segment of our population with the highest rates of dementia.^2^

## Methods

### Participants

**Figure 1.**
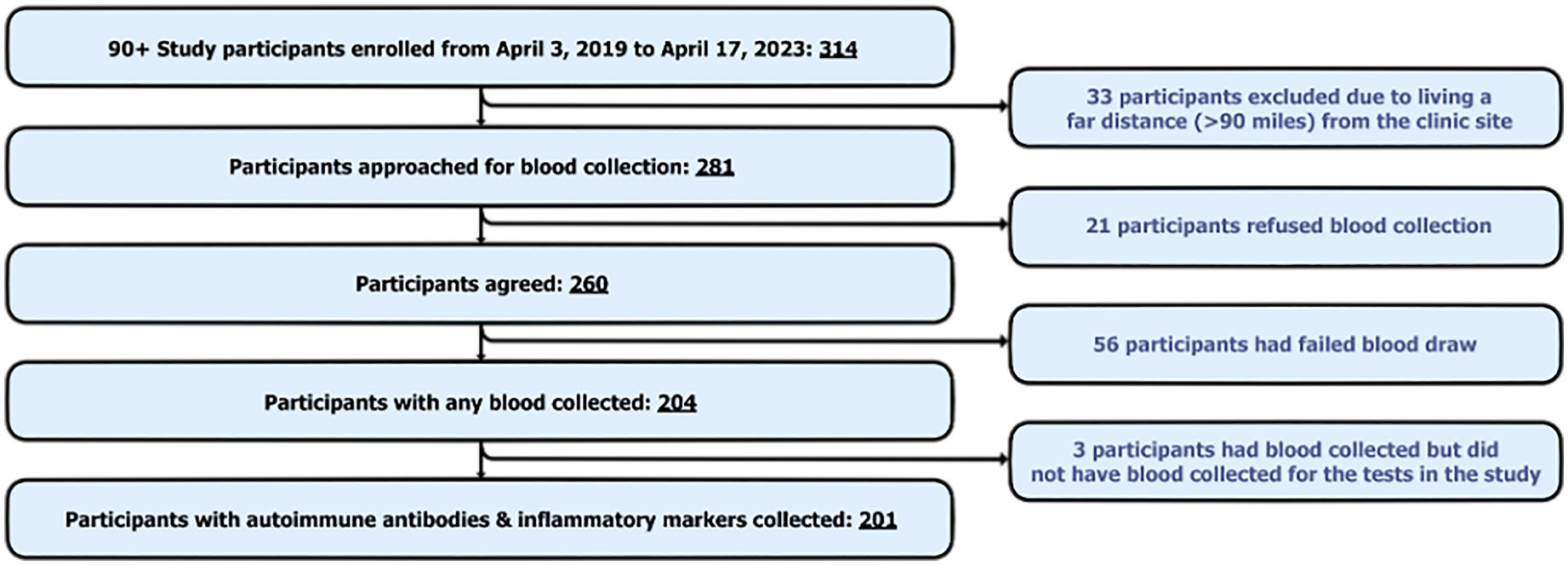
Flow chart depicting the sequential process of participant data collection. Analysis for the prevalence of autoimmune antibodies and inflammatory markers was conducted on blood samples from 201 individuals.

The participants in this study came from The 90+ Study, an ongoing longitudinal study of aging and dementia in age 90 + in Southern California. Figure 1 shows that out of 314 consecutive participants who participated in the study from April 3, 2019, the start date for acquiring samples for autoimmune antibodies and inflammatory markers, to April 17, 2023, 281 were approached for blood draw. Out of the approached participants, 204 had blood collected. Out of 204, 3 did not have the required blood volume to conduct the autoimmune antibody or inflammatory marker tests (Figure 1). To be included in this study, participants had to have at least one autoimmune antibody or inflammatory marker test result. All participants with test results had cognitive measures at the same visit as blood collection. Evaluations occur every 6 months until death and include self-reported medical history, medication use, neuropsychological testing, neurological examination, and blood collection. Neurological examination is performed by trained examiners (physicians and nurse practitioners) and includes the Functional Activities Questionnaire^13^ and Clinical Dementia Rating scale.^14^

The 90+ Study was approved by the Institutional Review Board of the University of California Irvine (approval no. 20012029). All participants or their surrogates provided written informed consent.

### Blood collection and testing

201 participants had serum test result for the autoimmune antibodies for at least one time point. Antibodies included antinuclear antibodies (ANA), antineutrophil cytoplasmic antibodies (ANCA), rheumatoid factor (RF), double-stranded DNA (DS-DNA), antithyroglobulin (ANTI-TG), and anti-thyroid peroxidase (ANTI-TPO). 136 of 201 participants had test results at multiple visits. All tests were done with standardized procedures. ANA, ANCA, and DS-DNA were detected by indirect immunofluorescence and considered present when the titer was >0. RF was measured using two methods of detection; the first using indirect immunofluorescence and second, implemented after December 29, 2021, using quantitative immunoturbidimetry. There are participants with RF results for both methods, hence there is an overlap in the number of participants within the first and second method groups*.* RF was considered present when the titer was >0 for method 1 and >0 IU/mL for method 2. For calculating the RF prevalence, positive results were extracted from both methods. ANTI-TG and ANTI-TPO were detected by a 2-step sandwich immunoassay. For ANTI-TG and ANTI-TPO, positivity criteria were >4 IU/mL and >34 IU/mL, respectively.

142 participants had result for the inflammatory markers, interleukin 6 (IL-6) and erythrocyte sedimentation rate (ESR), for at least one time point with most having plasma results at multiple visits. IL-6 was measured by chemiluminescence and ESR by Photometric Rheoscopy. For IL-6, concentrations greater than 6 pg/mL and for ESR values above 30 mm/hr in women and 20 mm/hr in men were considered elevated.

### Cognitive testing

A battery of neuropsychological tests^15,16^ was administered by trained testers that, among others, included the Mini-Mental State Examination (MMSE),^17^ Modified MMSE (3MS),^18^ and the California Verbal Learning Test (CVLT)^19^ long delay recall. A composite score for memory was calculated as the average standardized scores (mean/standard deviation) of the CVLT and memory measure of 3MS. Global cognitive score was calculated as the standardized score of 3MS or MMSE, if 3MS was not available.

### Image acquisition and analysis

A subset of participants had undergone an amyloid PET scan with Florbetapir (F-AV-45). Standardized uptake value ratio (SUVR) was calculated using the ratio of amyloid burden in a region of interest composed of posterior cingulate and precuneus over eroded white matter mask as reference. Based on prior postmortem validation study, amyloid PET scans with SUVR ≥ 0.76 were considered positive.^20^

### Statistical analysis

First, we describe the sequential process of participant data collection (Figure 1) and the characteristics of the participants (Table 1) and individuals who were not enrolled in the study (Supplemental Table 2). To compare the demographic features of those who enrolled and those excluded, and since the data was not normally distributed, Mann-Whitney U test was used for continuous variables, and Chi-square for categorical variables, *p* < 0.05 was considered significant. Next, for reporting the prevalence of autoimmune antibodies and inflammatory markers across all visits, we dichotomized the results through the positivity criteria presented in Table 2. We included all study participants with available test results for a given test as the denominator in the determination of prevalence. It is noteworthy that for each test, some participants had only one visit and the remaining had multiple visits. Prevalence was defined as having a positive or elevated result for at least one visit. For descriptive analysis, RF, ANTI-TG, ANTI-TPO, IL-6 and ESR concentrations were treated as continuous variables while titers of ANA, ANCA, and DS-DNA were treated as ordinal variables. For continuous variables mean ± standard deviation (SD) and for ordinal variables median ± interquartile range (IQR) were reported (Table 2). Within the subset of participants with multiple results (*n* = 136), we created three subgroups of participants who tested positive for (1) at least one visit, (2) ≥ 50% visits, and (3) all visits (Supplemental Table 1). These subgroups overlap as for instance, individuals with ≥ 50% positive results were also counted as those with at least one positive result (Supplemental Figure 1).

**Table 1.** Participant characteristics.

Total number of participants, *n* (%)	201 (100%)
Age at collection, years	
mean (SD)	94.8 (2.8)
median	94.4
[min, max]	[90.1, 106.6]
Female (%)	114 (56.7%)
Education, College Education or more, *n* (%)	116 (57.7%)
Race and ethnicity	
Asian, *n* (%)	7 (3.5%)
Black, *n* (%)	2 (1.0%)
White, *n* (%)	188 (93.5%)
Latinx, *n* (%)	4 (2.0%)
History of rheumatologic illness, *n* (%)	9 (4.5%)

The age at collection measures (i.e., mean, median, SD, median) were computed using age at every blood collection visit. Rheumatologic illnesses include rheumatoid arthritis, lupus, and scleroderma.

**Table 2.** Prevalence of autoimmune antibodies and inflammatory markers in all study participants.

Titer markers	Positivity criteria	Prevalence *n* (%)	Median for all results (IQR)	Median for positive results (SD)
ANA (*n* = 182)	≥1:40	89 (48.9)	0 (1:80)	1:80 (1:260)
ANCA (*n* = 179)	≥1:20	63 (35.2)	0 (0)	1:80 (1:120)
DS-DNA (*n* = 178)	≥1:10	12 (6.7)	0 (0)	1:80 (1:130)

Concentration markers	Positivity criteria	Prevalence *n* (%)	Mean for all results (SD)	Mean for positive results (SD)
RF (*n* = 178)	>0 IU/mL	49 (27.5)	0 IU/mL (0)	16 IU/mL (14)
ANTI-TG (*n* = 201)	>4 IU/mL	30 (14.9)	14.2 IU/mL (129)	116 IU/mL (367.2)
ANTI-TPO (*n* = 201)	>34 IU/mL	21 (10.5)	14.9 IU/mL (66.1)	163.8 IU/mL (187.6)
IL-6 (*n* = 128)	>6 pg/mL	88 (68.8)	10.2 pg/mL (18.5)	17.5 pg/mL (25.6)
ESR (*n* = 142) (F:89, M:53)	F: > 30 mm/hr	F: 42 (47.2)	F: 26.4 (16.5)	F: 46.3 (13.8)
	M: > 20 mm/hr	M: 29 (54.7)	M: 16.5 (21.5)	M: 36.2 (15.2)
		Overall: 71 (50.0)		

To address the dependency among observations resulting from multiple visits, we employed linear mixed-effects models. These models allowed us to investigate the association between repeated measures of blood markers and cognitive performance. Within these models, every test result was included regardless of whether a participant had one or multiple results. The first set of linear mixed effects models examined the relationship between the level of each serological marker (independent variable) and global cognitive and memory scores (dependent variables). The second set of linear mixed effects models examined this same relationship, but adjusted for amyloid burden, measured by SUVR, to assess the relationship between inflammation and autoimmunity markers that is independent of Alzheimer's disease.

We further examined whether the cognitive measure association between the biomarkers (autoimmune antibodies and inflammatory markers) and cognitive measure depends on the across-visit fluctuation in the biomarker results. To meaningfully define the fluctuation, we included in the analysis participants with more than one visit. The across-visit fluctuation was operationalized as the proportion of visits with positive biomarker results for a participant. As the number of visits for most of the biomarkers was four, we assigned 25% elevation of proportion positive as the measure for this variable. For each cognitive measure and each biomarker, we fitted a separate linear mixed effects model with response variable being the cognitive measure and the following regressors: the proportion of positive visits, gender, education, and time. A random intercept is included to account for the correlated outcomes across visits. A significant proportion positive term indicates that the increase in proportion of positive visits significantly affects the cognitive measure.

The variables were treated as continuous for every model. For RF, results for quantitative immunoturbidimetry method of measurement were used for the models. All models were adjusted for sex, education (college vs no college), age at each blood collection visit (centered at mean); and in the amyloid-adjusted model, an additional adjustment was made for the time between the most recent amyloid PET scan and closest blood collection visit.

Regression coefficients with 95% confidence interval is computed for all models. Analyses were performed through R, version 4.3.1 using the ‘lme4’ package. *p* < 0.05 was considered significant.

## Results

### Demographics

201 participants from The 90+ Study fulfilled the inclusion criterion of having at least one antibody and 142 had inflammatory marker results (Figure 1). Baseline characteristics are detailed in Table 1. The mean age at blood collection visits was 94.8 ± 2.8. 56.7% were women, and 57.1% had a college education. Most of the cohort participants were white (93.5%), and of non-Latinx ethnicity (98%), with few in other ethnoracial categories. Demographics of participants who did not have blood collected (*n* = 110) and thus were excluded from this study, are presented compared to those in our study (*n* = 201) in Supplemental Table 2. Participants who did not have blood collected (*n* = 110) were on average older, more were female, and more had cognitive impairment when compared to those in this study (*n* = 201) (Supplemental Table 2).

### Distribution of autoimmune antibodies and inflammatory markers

For reporting prevalence of inflammatory markers and autoantibodies, each individual with at least one positive result was considered positive. 70.2% of participants had at least one positive autoimmune antibody (141/201) and 76.8% of participants had at least one elevated inflammatory marker (109/142). The prevalence of individual autoimmune antibodies in our study cohort were 48.9% for ANA (*n* = 182), 35.2% for ANCA (*n* = 179), and 27.5% for RF (*n* = 178). Regarding markers of inflammation, we observed elevated IL-6 (*n* = 128) in 68.8% and ESR (*n* = 142) in 50% of participants (Table 2).

We also investigated the frequency of positive results in participants with multiple measurements (*n* = 136 for antibodies and *n* = 103 for inflammatory markers). The mean number of visits was 4 for most of the autoimmune antibodies and inflammatory markers (Supplemental Table 1). In this subset of participants, 79.4% had at least one autoimmune antibody (108/136) and 81.5% had at least one raised inflammatory marker (84/103). The prevalence of antibodies was 56% for ANA (*n* = 116), 40% for ANCA (*n* = 115) and 33.6% for RF (*n* = 115). Regarding markers of inflammation, we observed elevated IL-6 in 69.3% (61/81) and ESR (*n*) in 49.5% (71/142) of participants. Prevalence of positive results in ≥50% of visits were 38% for ANA, 27% for ANCA, 51% for IL-6 and 35.9% for ESR. Prevalence of positive results for all visits were 14% for ANA, 10% for ANCA, 15% for IL-6 and 20% for ESR (Supplemental Table 1).

### Association of higher levels of autoimmune antibodies and inflammatory markers with lower cognitive performance

**Figure 2.**
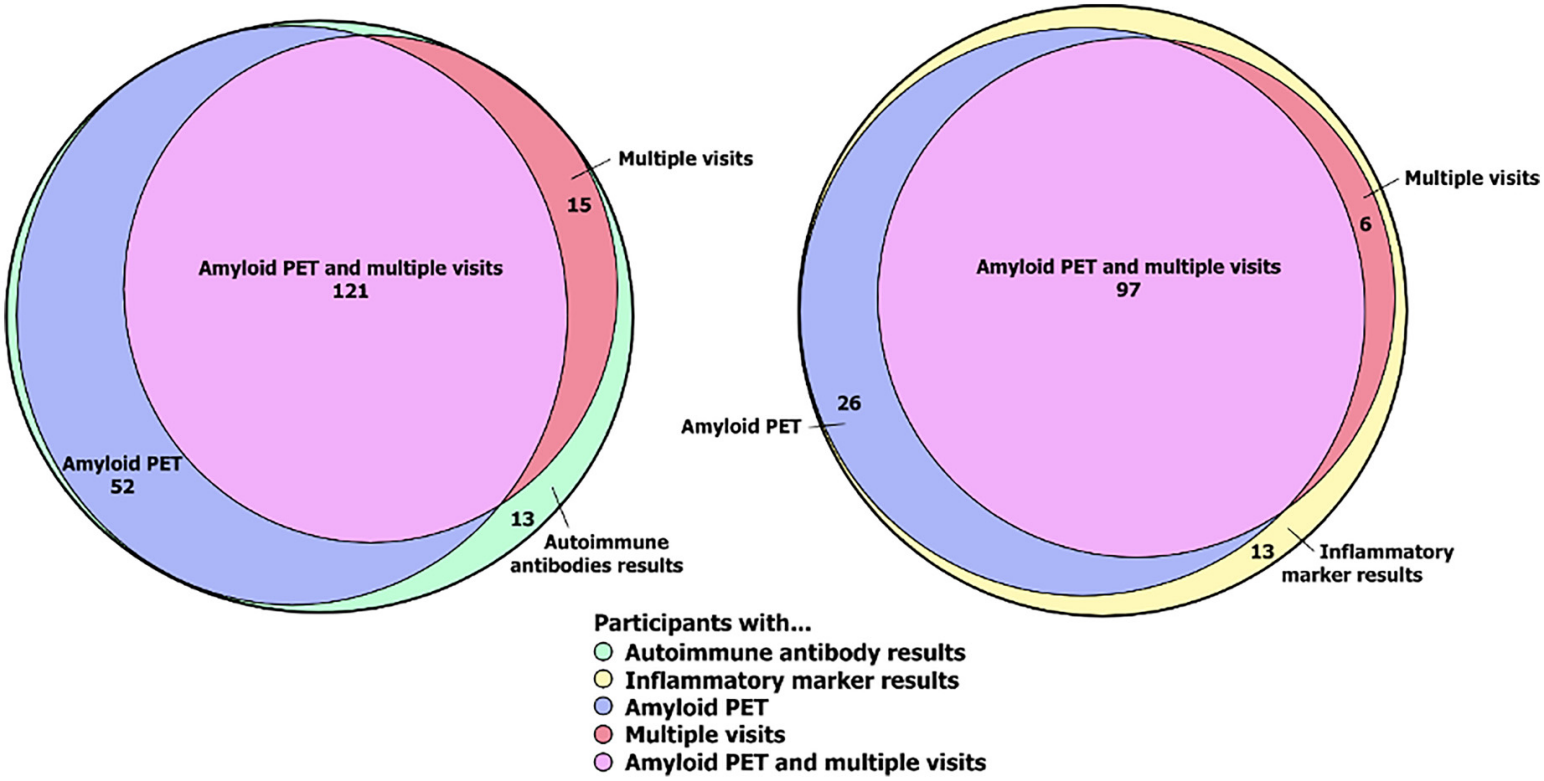
Euler diagram of the composition of the study population. Diagram depicts measures of autoimmune antibodies and inflammatory markers. Of the 201 individuals who had measures of autoimmune antibodies, 173 had amyloid PET imaging and 136 had multiple antibody measures. Of the multiple measurement subset, 121 had amyloid PET. From the 201 total participants, inflammatory markers were measures in 142 individuals. 123 of these individuals had amyloid PET, and 103 had multiple measurements. Of the multiple measurement group, 97 had amyloid PET.

To explore the association between autoimmune antibodies, inflammatory markers (independent variables), and cognitive performance (dependent variable), we used linear mixed effects models, to account for repeated measurements of test results in participants with multiple visits. We adjusted for sex, education (college vs no college), and age (aligned at mean). Higher titers of ANCA (*p* = 0.04) and higher levels of IL-6 (*p* = 0.01) and ESR (*p* = 0.01) were significantly associated with worse global cognition. For ANCA, every 1:40 increase in titer was associated with a 0.03-point decrease in global cognitive score. For IL-6, an increase of 10 pg/mL was associated with a 0.05-point decrease in global cognitive score. For ESR, every 10 mm/hour increase was associated with a 0.07-point decrease in global cognitive score. We further adjusted for amyloid burden in participants with available PET scan (*n* = 173 for autoimmune antibodies and *n* = 123 for inflammatory markers) (Figure 2). In this model the coefficients for most variables, including ANCA, IL-6, and ESR remained virtually the same, with the association between ANCA, IL-6, and ESR with global cognitive score holding significance (Table 3). Regarding the association of examined markers with memory performance, the association of higher levels of ANCA (*p* = 0.07) and IL-6 (*p* = 0.09) with lower standardized memory scores trended toward significance (Table 3).

**Table 3.** Associations between autoimmune antibodies and inflammatory markers with cognitive performance in all study participants.

	Global cognitive score				Memory score			
	Not adjusted for amyloid		Adjusted for amyloid		Not adjusted for amyloid		Adjusted for amyloid	
	Estimates (95% CI)	*p*	Estimates (95% CI)	*p*	Estimates (95% CI)	*p*	Estimates (95% CI)	*p*
ANA	0.00006 (−0.00003, 0.00015)	0.20	0.00006 (−0.00003, 0.00015)	0.18	0.00002 (−0.00007, 0.0001)	0.72	0.00002 (−0.00007, 0.0001)	0.65
ANCA	−0.0008 (−0.0015, −0.00003)	0.04*	−0.0009 (−0.00164, −0.00008)	0.03*	−0.0007 (−0.001, 0.00004)	0.07	−0.0005 (−0.001, 0.0002)	0.18
RF	−0.0006 (−0.00583, 0.00466)	0.83	−0.0003 (−0.0057, 0.00506)	0.91	−0.004 (−0.008, 0.001)	0.13	−0.004 (−0.008, 0.001)	0.13
ANTI-TG	0.0002 (−0.00053, 0.00092)	0.59	0.0003 (−0.0004, 0.00093)	0.44	0.0002 (−0.0003, 0.0008)	0.41	0.0003 (−0.0002, 0.0009)	0.31
DS-DNA	−0.0005 (−0.00137, 0.00034)	0.24	−0.00006 (−0.00105, 0.00093)	0.91	−0.00005 (−0.0008, 0.0007)	0.91	0.0003 (−0.0006, 0.001)	0.49
ANTI-TPO	0.0004 (−0.00044, 0.0013)	0.59	0.00005 (−0.00037, 0.00134)	0.26	0.0002 (−0.0006, 0.0009)	0.69	0.0002 (−0.0005, 0.001)	0.56
IL-6	−0.005 (−0.00862, −0.00108)	0.01*	0.005 (−0.00861, −0.0011)	0.01*	−0.003 (−0.006, 0.0004)	0.09	−0.003 (−0.006, 0.0006)	0.11
ESR	−0.007 (−0.01117, −0.00183)	0.01*	−0.008 (−0.01238, −0.00287)	0.007*	−0.003 (−0.006, 0.001)	0.25	−0.004 (−0.008, 0.0009)	0.12

Estimates and *p-values* from the linear mixed effect models. All models adjusted for age (aligned at mean), sex, and education (college versus no college), as well as incorporate standardized values for global cognitive and memory score. First model did not account for amyloid burden on PET imaging, while the second model adjusted for amyloid burden (SUVR). *0.05 < *p*, CI: confidence interval.

To examine whether proportion of visits with positive/elevated results was associated with cognitive performance, we used a linear mixed effects model. For IL-6, a 25% increase in the proportion of visits with elevated levels was significantly associated with a 0.143-point decrease in global cognitive score (95% CI: (.0057, .2805), *p* = 0.044) and 0.155-point decrease in memory score (95% CI: (0.0257, 0.2842), *p* = 0.021). After adjusting for amyloid, the associations remained significant. Although not present in the model before adjusting for amyloid, we found for ANCA that a 25% increase in proportion positive was significantly associated with a 0.127-point decrease in memory score (95% CI: (0.014, 0.241) *p* = 0.03) after the amyloid adjustment.

## Discussion

In this study, we investigated the frequency of serological markers of autoimmunity and inflammation in individuals aged 90 + and investigated their association with measures of global cognition and memory. This study revealed that these markers were highly prevalent in this age group and associated with lower cognitive abilities. Specifically, we found that higher levels of ANCA, IL-6, and ESR were associated with lower global cognitive scores. Having abnormal results in a higher proportion of the visits for ANCA and IL-6 were also associated with worse cognitive outcomes. Moreover, these associations appeared to be independent of Alzheimer's disease pathology measured by amyloid PET scan.

Previous studies in younger age groups have demonstrated the prevalence of autoimmune antibodies, such as ANA, and inflammatory markers, like IL-6, without the presence of any specific autoimmune or inflammatory disease diagnosis in the aging population.^9,10,12^ In a sample of participants aged 60–69, 70–79, and 80+, the prevalence of ANA positivity increased stepwise, with 18.5%, 21.9%, and 37.5% testing positive, respectively.^9^ The prevalence of ANA positivity in our study's older than 90 cohort was 48.9%, illustrating continued increase in ANA prevalence in the tenth decade of life. Similarly for the inflammatory marker IL-6, a study comprised of younger-old participants, aged 65+, found that 21.6% had elevated IL-6 levelsClick or tap here to enter text.,^10^ while our study's oldest cohort had 68.8%. Studies on other autoimmune antibodies and inflammatory markers also suggest that advanced age is a risk factor for higher prevalence of these markers.^21,22^ We postulate that the increased frequency of autoimmune antibodies and inflammatory markers, especially in the 10^th^ and 11^th^ decade of life, results from an accumulation of genetic mutations and environmental triggers that increase immunosenescence, the age-related decline of the immune system. This decline in immune function, particularly the reduced ability to differentiate between foreign and self-antigens, may lead to increased production of autoimmune and inflammatory markers.^23,24^ Contributing factors may include altered B cell function, a decrease in naïve T-cells, and an increase in proinflammatory cytokines,^23^ all part of a complex mechanism exacerbating the autoimmune response. Moreover, environmental triggers, such as recurring viral infections, could induce the production of natural autoantibodies like ANA or ANCA in older individuals.^24,25^ For instance, a study found a significant correlation between ANA levels and influenza-specific antibodies, indicating that natural autoimmune antibodies might play a role in the increased production of autoantibodies, like ANA and ANCA, in elderly individuals 25.

Another important finding of our study was the association between higher levels of autoimmune antibody, ANCA and markers of inflammation, IL-6 and ESR, with lower global cognition (Table 3). These associations were observed in our entire cohort and having more consistent abnormal results for ANCA and IL-6 was associated with worse cognitive performance. Altogether, these findings reveal the deleterious effects of presence of markers of inflammation and autoimmunity on cognitive performance especially when the antibodies are more consistently present, suggesting that temporary increase of these markers such as in the context of infection, is not associated with cognitive outcome. A more consistent increase in autoantibodies and inflammatory markers in the context of autoimmunity and chronic inflammation could be more substantially related to cognitive performance. Senescent immune cells could enhance proinflammatory effects and promote neuroinflammation, implicating a potential role for immunity in neurodegenerative mechanisms.^23,24^ It is plausible that serological markers of autoimmunity and inflammation are surrogate markers of neuroinflammation that in turn lead to neurodegeneration. Another mechanism possibly explaining the significant association between immune dysfunction and lower cognitive scores is the increased permeability of the blood-brain barrier (BBB). The accumulation of autoimmune antibodies and inflammatory markers, as observed in autoimmune encephalitis, may compromise the integrity of endothelial cells. This allows various immune and inflammatory molecules into the brain, potentially inducing neuroinflammation.^26^ Furthermore, the disruption of the BBB and the influx of antibodies and other molecules could lead to excitotoxicity, neurotransmitter imbalances, and neuronal cell death.^26^ IL-6 accumulation may also promote oxidative stress, reducing nitric oxide availability and increasing NADPH oxidase-derived superoxide, resulting in neuronal damage and cognitive impairment 12.

Intriguingly, the observed associations between the blood markers of inflammation and autoimmunity and cognitive outcomes were not affected by adjusting for amyloid burden. This suggests that the relationship between autoimmunity and inflammation with lower cognitive performance could be explained by mechanisms other than Alzheimer's disease (AD) neuropathologic change. In other words, either non-AD neuropathologies are mediating this relationship or inflammation and autoimmunity can directly lead to cognitive impairment. Therefore, examining the association of inflammation and autoimmunity with non-AD pathologic changes is warranted. Our finding is contrary to some studies which have suggested the role of inflammation is through its contribution to amyloid accumulation and Alzheimer's pathology in old individuals.^27,28^ In the 90 + age group, non-Alzheimer's neuropathologies become more common and can frequently mimic Alzheimer's disease.^26^ Limbic-predominant age-related TDP-43 encephalopathy neuropathologic change (LATE-NC) and hippocampal sclerosis of aging (HS-A) are examples of these non-Alzheimer's pathologies.^4,29^ Putative relationship of these pathologic changes and inflammation and autoimmunity is worth further investigation in diverse samples of aged 90 + cohorts with available neuropathology.

This study has certain limitations. Our participants were almost exclusively from a white background, which limits the generalizability of our findings to other racial and ethnic groups. This was due to our sample being from one geographic region of the United States, where most of the elderly population is from a white background. There was a disparity between participants who did not have blood collected and those included in our study for age, sex, and cognitive diagnosis at last visit. In those included in our study, more were cognitively normal at last visit which may have led them to participate in more aspects of The 90+ Study, such as the blood draw. Also, due to the advanced age of study participants and frequent visual problems, memory assessment in this cohort is limited to a few verbal memory tests that might limit the observed associations between memory and other measures. Addressing these limitations in future studies could provide more comprehensive insights. Additionally, incorporating measurements of other inflammation and oxidative stress markers might further enhance our understanding of these complex relationships. The advantages of this study include the unique age group of participants, serological measures of autoimmunity and inflammation at multiple time points, and availability of amyloid PET for many participants.

### Conclusion

Our study highlights the high frequency of autoimmune antibodies and inflammation markers in individuals aged 90 and above. Notably, we observed an inverse relationship between autoantibody ANCA and inflammatory markers, ESR and IL-6, and global cognitive score. Future research is warranted to explore the impact of autoimmune and inflammatory processes in non-Alzheimer's disease dementias.

## Supplemental Material

sj-docx-1-alz-10.1177_13872877251365560 - Supplemental material for Autoimmune antibodies and systemic inflammatory markers are prevalent and associated with cognition in individuals aged 90+ Supplemental material, sj-docx-1-alz-10.1177_13872877251365560 for Autoimmune antibodies and systemic inflammatory markers are prevalent and associated with cognition in individuals aged 90+ by Ghasem Farahmand, Anne-Marie C Leiby, Jiaxin Yu, Aanan Ramanathan, Rojan Javaheri, Claudia H Kawas, Davis C Woodworth, Maria M Corrada, Tianchen Qian and S Ahmad Sajjadi in Journal of Alzheimer's Disease
