# Supplementary material for: Autoimmune antibodies and systemic inflammatory markers are prevalent and associated with cognition in individuals aged 90+
Source: J Alzheimers Dis. 2025 Aug 8;107(3):1217–25. doi: 10.1177/13872877251365560 (PMC12449596; doi:10.1177/13872877251365560)
Supplement: sj-docx-1-alz-10.1177_13872877251365560 - Supplemental material for Autoimmune antibodies and systemic inflammatory markers are prevalent and associated with cognition in individuals aged 90+ [file sj-docx-1-alz-10.1177_13872877251365560.docx]

**Supplemental Material**

**Autoimmune antibodies and systemic inflammatory markers are prevalent and associated with cognition in individuals aged 90+**

| Supplemental Table 1. Prevalence of autoimmune antibodies and inflammatory markers in a subset of participants with multiple measures | | | | |
| --- | --- | --- | --- | --- |
| Marker | **Number of visits**  mean [range] | **Positive for at least one visit**  *n* (%) | **Positive for ≥ 50% visits**  *n* (%) | **Positive for all visits**  *n* (%) |
| ANA (*n*=116) | 4 [2,6] | 65 (56) | 44 (37.9) | 16 (13.8) |
| ANCA (*n*=115) | 3.9 [2,6] | 46 (40) | 31 (27) | 11 (9.6) |
| RF (*n*=115) | 3.9 [2,6] | 38 (33.0) | 20 (17.4) | 10 (8.7) |
| ANTI-TG (*n*=136) | 4.2 [2,7] | 22 (16.2) | 14 (10.3) | 12 (8.8) |
| DS-DNA (*n*=115) | 3.9 [2,6] | 10 (8.7) | 5 (4.4) | 0 (0) |
| ANTI-TPO (*n*=135) | 4.3 [2,7] | 15 (11.1) | 12 (8.9) | 8 (5.9) |
| IL-6 (*n*=88) | 3.0 [2,4] | 61 (69.3) | 45 (51.1) | 13 (14.8) |
| ESR (*n*=103) | 3.3 [2,5] | 51 (49.5) | 37 (35.9) | 20 (19.4) |

The number of participants that meet the criteria for each category are represented by *n.* When (%) is calculated, the denominator is the total number of participants with multiple measures (*N*).

| Supplemental Table 2. Comparison of characteristics between participants with blood for autoimmune antibodies and inflammatory markers vs participants with no blood | | |
| --- | --- | --- |
|  | **Participants with blood for autoimmune antibodies and inflammatory markers** | **Participants with no blood** |
| Total number of participants, *n* (%) | 201 (100%) | 110 (100%) |
| Age at collection, years  mean (SD)  median  [min, max] | 94.8 (2.8)  94.4 *  [90.1, 106.6] | 95.5 (3.4)  95.1  [90, 110.5] |
| Female (%) | 114 (56.7%) * | 78 (70.9%) |
| Education, College Education or more, *n* (%) | 116 (57.7%) | 56 (50.9%) |
| Race/ Ethnicity  Asian, *n* (%)  Black, *n* (%)  White, *n* (%)  Latinx, *n* (%) | 7 (3.5%)  2 (1.0%)  188 (93.5%)  4 (2.0%) | 4 (3.6%)  -  105 (95.5%)  1 (0.9%) |
| History of rheumatologic illness, *n* (%) | 9 (4.5%) | 4 (3.6%) |
| Cognitive diagnosis at first visit, *n* (%) | CN: 106 (52.7%)  CIND: 74 (36.8%)  DEM: 21 (10.5%) | CN*: 63 (65.0%) ‡  CIND*: 25 (25.8%) ‡DEM*: 9 (9.3%) ‡ |
| Cognitive diagnosis at last visit, *n* (%) | CN: 95 (47.3%) *  CIND: 69 (34.3%) *  DEM: 38 (18.9%) * | CN: 27 (31.8%) †  CIND: 25 (29.4%) †  DEM: 30 (35.3%) † |

The age at collection measures (i.e., mean, median, SD, median) were computed using age at every blood collection visit. *There is a statistically significant (p<0.05) difference between participants with blood (n=201) and with no blood (n=110) for age, sex, and cognitive diagnosis at last visit. ‡97 out of the 110 participants with no blood had cognitive diagnoses at first visit and †85 out of the 110 had cognitive diagnoses at last visit. Participants had missing cognitive diagnoses when their visit was by telephone or completed by their assigned informant. For some socially distanced visits, cognitive diagnosis was also missing. CN: cognitively normal; CIND: cognitive impairment, no dementia; DEM: dementia


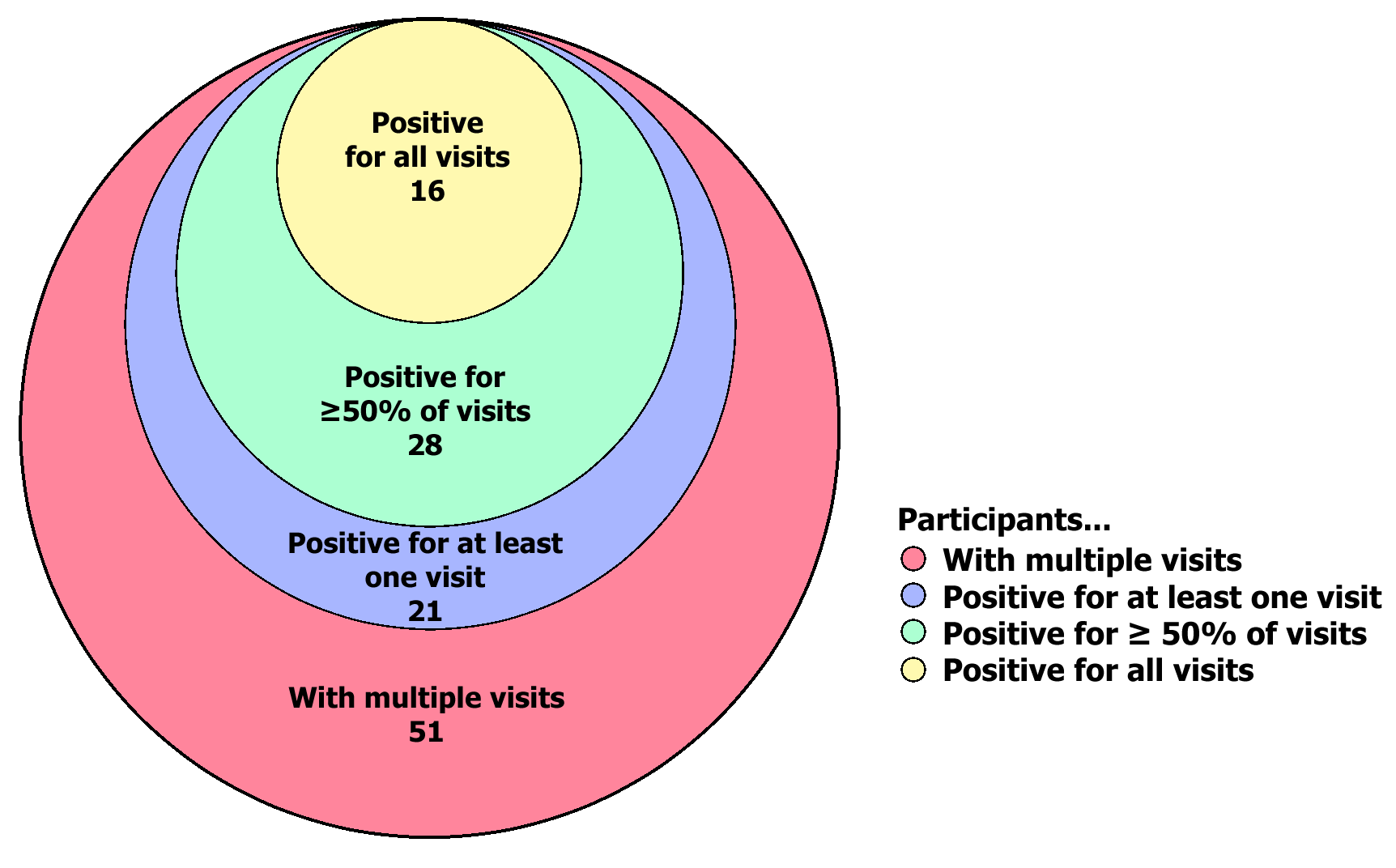


**Supplemental Figure 1.** Euler diagram demonstrating the number of participants with multiple visits for ANA and the proportion of those with different frequencies of ANA positivity across visits (positive for at least one visit, positive for > 50% of visits, and positive for all visits).
